# Navigation by Induction-Based Magnetoreception in Elasmobranch Fishes

**DOI:** 10.1155/2009/380976

**Published:** 2009-10-18

**Authors:** T. C. A. Molteno, W. L. Kennedy

**Affiliations:** Department of Physics, University of Otago, P.O. Box 56, Dunedin 9016, New Zealand

## Abstract

A quantitative frequency-domain model of induction-based magnetoreception is presented for elasmobranch fishes. We show that orientation with respect to the geomagnetic field can be determined by synchronous detection of electrosensory signals at harmonics of the vestibular frequency. The sensitivity required for this compass-sense mechanism is shown to be less than that known from behavioral experiments. Recent attached-magnet experiments have called into doubt the induction-based mechanism for magnetoreception. We show that the use of attached magnets would interfere with an induction-based mechanism unless relative movement between the electrosensory system and the attached magnet is less than 100 *μ*m. This suggests that further experiments may be required to eliminate induction as a basis for magnetoreception.

## 1. Introduction

Behavioral experiments show that elasmobranch fishes (sharks, skates, and rays) can detect changes in the geomagnetic field [1–3], and studies of migration [4, 5] give strong evidence that several species can navigate over long distances in environments where the geomagnetic field is the only plausible reference [6]. Both direct magnetoreception and induction-based electroreception have been proposed as mechanisms for this ability to orient to the geomagnetic field or “compass sense”.

The direct magnetoreception mechanism [7] assumes the existence of magnetite-based *magnetoreceptors* whose primary function is to measure the geomagnetic field for the purposes of navigation. The locus or mode of transduction for this magnetic sense is still the subject of some debate (see, e.g., Johnsen and Lohmann and others [7–9]).

The electrosensory mechanism [10, 11] proposes that the orientation to the geomagnetic field is primarily achieved by magnetic induction; movement through the geomagnetic field induces currents in the electrosensory system, that are then used to achieve a compass sense. Behavioral experiments by Hodson [12] and others [8] using attached magnets have cast doubt on the electrosensory mechanism. Bar magnets, inserted into the nasal cavity of the short-tailed stringray, *Dasyatis brevicaudata*, impaired ability to detect magnetic field gradients. A magnetic field that is stationary with respect to the electrosensory system should have no effect on a mechanism based on magnetic induction. As the body of a ray is flexible, other authors have suggested that movement of the body with respect to the magnet might have impaired an induction-based system [7].

In this paper we begin with an analysis of induction-based mechanisms [11] for magnetoreception and show directly how the amplitudes of electrosensory signals at harmonics of the vestibular signals can be used to achieve a compass sense. An analysis of the magnitudes of these harmonics shows that the signals could be detected by the elasmobranch electrosensory system. We then show, using a simplified body-flexing model of a swimming fish, that relative movement of an attached magnet would impair an induction-based mechanism, unless strict criteria are met.

## 2. Induction-Based Magnetoreception

Without ocean current, the only motion through the geomagnetic field is caused by the fish. Charged particles in the electrosensory system experience forces due to motion (*v*_*h*_^*w*^) through the geomagnetic field (*B*) and any local electric fields (*E*) within the electrosensory system. These Lorentz forces *F*_*L*_ are described by

(1) $$F_{L}=q\left(E+v_{h}^{w}\times B\right),$$

where *q* is the charge on each charge carrier in the ampullae, and × is the usual vector cross-product. Charges will move as a result of this force and this leads to induced electric fields (even if none were present before). An equilibrium is reached when these forces add to zero, that is,

(2) $$E=-v_{h}^{w}\times B.$$

We use an earth frame with the *y*-axis along magnetic north. The components of the geomagnetic field in the earth frame are

(3) $$B=\left(\begin{array}{c} 0\\ B_{y}\\ B_{z} \end{array}\right).$$

If the fish is swimming with a heading Θ, a simple model assumes that its swimming follows a sinusiodal path [11] and that the angle between the mean fish path and the head oscillates as *α*sin(*ωt*), where *ω* is the oscillation frequency of the head during swimming, and *α* is the angular head oscillation amplitude during swimming. The frequency *ω* is the “vestibular frequency” for the swimming motion. The angle *ϕ* between the head and geomagnetic north is then

(4) ϕ=Θ+αsin(ωt).

In the fish frame, the geomagnetic field appears rotated by an angle −*ϕ* about the *z* axis:

(5) $$B^{f}=R_{z}\left(-\phi \right)B=\left(\begin{array}{c} B_{y}\sin\left(-\theta -\alpha \sin\left(\omega t\right)\right)\\ B_{y}\cos\;\left(-\theta -\alpha \sin\left(\omega t\right)\right)\\ B_{z} \end{array}\right).$$

The equilibrium electric field due to induction will then be

(6) $$\begin{array}{c} E^{f}=-v_{h}^{f}\times B^{f} \end{array}$$

or

(7) $$\begin{array}{c} E^{f}=\left(\begin{array}{c} -B_{z}v_{y}^{f}\\ 0\\ -B_{y}v_{y}^{f}\sin\left(\theta +\alpha \sin\left(\omega t\right)\right) \end{array}\right) \end{array},$$

and the *z*-component of the electric field can be expanded to

(8) $$\begin{array}{cc} E_{z} & =-B_{y}v_{y}^{f}\cos\;\left(\theta \right)\sin\left(\alpha \sin\left(\omega t\right)\right)\\ & \;-B_{y}v_{y}^{f}\cos\;\left(\alpha \sin\left(\omega t\right)\right)\sin\left(\theta \right). \end{array}$$

### 2.1. Series Expansion

The Jacobi-Anger expansion [13] for *e* ^*ix*sin(*ωt*)^ allows us to expand sin(*α*sin(*ωt*)) and cos (*α*sin(*ωt*)) as series expansions:

(9) $$\begin{array}{c} \begin{array}{c} \sin\left(\alpha \sin\left(\omega t\right)\right)=2\overset{\infty }{\underset{n=0}{\sum }}J_{2n+1}\left(\alpha \right)\sin\left(\left(2n+1\right)\omega t\right) \end{array},\\ \begin{array}{c} \cos\;\left(\alpha \sin\left(\omega t\right)\right)=J_{0}\left(\alpha \right)+2\overset{\infty }{\underset{n=1}{\sum }}J_{2n}\left(\alpha \right)\cos\;\left(2n\omega t\right) \end{array}, \end{array}$$

where *J*_*n*_(*α*) are the Bessel functions of the first kind:

(10) $$J_{n}\left(\alpha \right)\equiv \overset{\infty }{\underset{l=0}{\sum }}\frac{{\left(-1\right)}^{l}}{2^{2l+n}l!{\left(n+l\right)}^{!}}\alpha^{2l+n}.$$

Applying the Jacobi-Anger expansion ((9)) to the expressions for the *x*-and *z*-components of (8) allows us to express the receptor electric field as a sum of sinusoidal functions, that is,

(11) $$\begin{array}{cc} & E_{x}=-B_{z}v_{y}^{f}, \end{array}$$

(12) $$\begin{array}{cc} & E_{z}=-B_{y}v_{y}^{f}[J_{0}\left(\alpha \right)\sin\Theta +2J_{1}\left(\alpha \right)\cos\;\Theta \sin\left(\omega t\right)\\ & \;\;\;\;+2J_{2}\left(\alpha \right)\sin\Theta \cos\;\left(2\omega t\right)\\ & \;\;\;\;+2J_{3}\left(\alpha \right)\cos\;\Theta \sin\left(3\omega t\right)\ldots ]. \end{array}$$

The *z*-component can be expressed as a sum of oscillating terms that are at integer multiples (harmonics) of the vestibular frequency *ω*:

(13) $$E_{z}=A_{z}^{0}+A_{z}^{\omega }\sin\left(\omega t\right)+A_{z}^{2\omega }\cos\;\left(2\omega t\right)+\cdots ,$$

where the DC term, *A*_*z*_^0^, is *B*_*y*_ *v*_*y*_^*f*^ *J*_0_(*α*)sinΘ, the amplitude of the frequency component at the vestibular frequency *ω*, *A*_*z*_^*ω*^, is

(14) $$\begin{array}{c} A_{z}^{\omega }=2B_{y}v_{y}^{f}J_{1}\left(\alpha \right)\cos\;\Theta , \end{array}$$

the amplitude of the second harmonic of the vestibular frequency, *A*_*z*_^2*ω*^, is

(15) $$A_{z}^{2\omega }=2B_{y}v_{y}^{f}J_{2}\left(\alpha \right)\sin\Theta ,$$

and the amplitude of the third harmonic of the vestibular frequency, *A*_*z*_^3*ω*^, is

(16) $$\begin{array}{c} A_{z}^{3\omega }=2B_{y}v_{y}^{f}J_{3}\left(\alpha \right)\cos\;\Theta \end{array}.$$

## 3. Signal Amplitudes

There is a considerable body of work on the sensitivity of elasmobranch electric senses (see Peters et al. [14] for an overview). Murray [3] shows that the * Ampullae of Lorenzini* are sensitive to electric fields. When the stimulus is applied as a voltage gradient in the water overlying the ampullae, the threshold for the most sensitive units is 100 *μ*Vm^−1^. The work of Kalmijn [15] showed that external fields as small as 2 *μ*Vm^−1^ could induce orienting behavior in the smooth dogfish, * Mustelus canis*.

More recently, Kajiura and Holland [16] found a median behavioral-response threshold for scalloped hammerhead sharks, * Sphyrna lewini*, of 2.5 *μ*Vm^−1^, and sandbar sharks, * Carcharhinus plumbeus*, of 3.5 *μ*Vm^−1^, and a minimum behavioral-response of ≈0.05 *μ*Vm^−1^ in both species. Peters et al. [14] concluded that angular swimming movements can induce stimuli that have a detection threshold of 0.1 *μ*Vm^−1^.

As with most sensory systems, electrosensory neurons do not respond to constant stimuli. Tricas and New [17] measured the frequency response of the afferent neurons in the round stingray, * Urolophus halleri*, and showed that these are sensitive to frequencies between approximately 0.1 Hz and 10 Hz.

Applying the typical parameters shown in Table 1 to (14), (15), and (16) gives the amplitude of the electric fields at the first three harmonics of the vestibular frequency:

(17) $$\begin{array}{cc} A_{z}^{0} & \approx 23.4\sin\Theta \;\mu \text{V}{\text{m}}^{-1},\\ A_{z}^{\omega } & \approx 12.1\cos\;\Theta \;\mu \text{V}{\text{m}}^{-1},\\ A_{z}^{2\omega } & \approx 1.53\sin\Theta \;\mu \text{V}{\text{m}}^{-1},\\ A_{z}^{3\omega } & \approx 0.13\cos\;\Theta \;\mu \text{V}{\text{m}}^{-1}. \end{array}$$

Both the amplitudes at the first harmonic, *A* ^*ω*^, and the second harmonic amplitudes, *A* ^2*ω*^, exceed the detection thresholds described above.

## 4. An Induction-Based Compass Sense

As the electroreceptors are not sensitive to DC stimuli [17], a compass sense should not use the constant *A*_*z*_^0^ term. Using the other harmonics, a compass direction can be found by comparing the amplitudes of the *z*-component of the electric field at the fundamental, *A* ^*ω*^, and second harmonic, *A* ^2*ω*^, of the vestibular frequency *ω*. Choosing the *z*-component, the ratio, Γ_*z*_, of these two amplitudes can be expressed in terms of the heading Θ as

(18) $$\Gamma_{z}\equiv \frac{A_{\text{z}}^{2\omega }}{A_{z}^{\omega }}=\frac{J_{2}\left(\alpha \right)}{J_{1}\left(\alpha \right)}\tan\left(\Theta \right).$$

**Figure 3:**
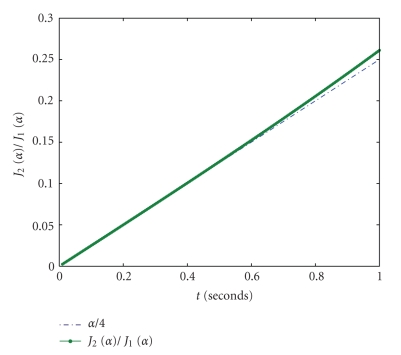
The Bessel function ratio *J*_2_(*α*)/*J*_1_(*α*) for swimming modulation amplitudes, *α*, between 0.0 and 1.0 radians (3–60 degrees). This ratio is well approximated by *α*/4 over this range.

**Figure 4:**
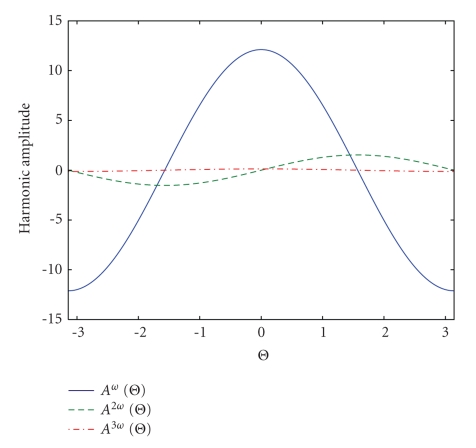
A plot of the harmonic amplitudes as a function of heading angle Θ (radians) for typical parameters.

The ratio Γ has many desirable properties as a compass sense. In particular, it is independent of the swimming speed |*v* ^*w*^| and the strength of the geomagnetic field. The factor *J*_2_(*α*)/*J*_1_(*α*) ≈ *α*/4 depends on the swimming modulation amplitude *α*. Figure 3 shows how the Bessel functions ratio changes for different swimming modulation amplitudes, *α*, between 0.05 and 0.5 radians. Figure 4 shows the harmonic amplitudes as a function of heading angle Θ (radians) for the typical parameters shown in Table 1.

Equation (18) clarifies how the compass sense, first suggested by Kalmijn [10] and refined by Paulin [11], could be achieved using electrosensory signals at harmonics of the vestibular frequency.

An advantage of this model is that it provides a plausible cognitive mechanism for long-distance magnetic navigation. Various models for navigation have been proposed (see e.g., Walker et al. [18]) including the following of magnetic anomalies in the ocean floor. The induction-based compass sense described here could enable an animal to travel long distances in the same direction by holding a constant ratio of *A*_*z*_^2*ω*^ to *A*_*z*_^*ω*^. In addition, the animal needs simply change the phase of one component by 180 degrees to travel on the return journey. This mechanism is relatively simple from a cognitive standpoint as it avoids the requirement for complex “maps” of magnetic anomalies that would be needed for long distance navigation.

## 5. Effects of Attached Magnets

A permanent magnet attached to a swimming elasmobranch, if not moving relative to the electrosensory system, will not create any induced electric field and should not interfere with an induction-based mechanism for magnetoreception. However experiments have shown [12] that placement of a permanent magnet in the nasal cavity of a short-tailed stringray, *Dasyatis brevicaudata*, does interfere with the ray's ability to sense magnetic field gradients.

Following the treatment in the previous sections, we can estimate the upper limits on relative movement between the magnet and the sensory system before the signals would exceed those from movement through the geomagnetic field.

**Figure 5:**
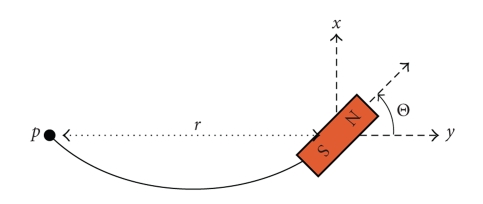
Simplified swimming model of a flexible fish with an attached magnet. The electroreceptor is located at *p*.

A simple model of a flexing swimming fish (see Figure 5), has a distance *r* between the electoreceptor and the magnet that varies as *r*/*r*_0_ ≈ 1 − (Θ^2^/6) as the angle Θ of the magnet changes during the swimming cycle. Here *r*_0_ is the distance when the body is straight, and *α* is an angular modulation amplitude for the flexing body.

If we assume that for regular swimming motion Θ = *α*sin*ωt*, then the relative velocity along the *y*-axis between the electroreceptor at point *p* and the magnet is

(19) $$\begin{array}{c} v=\left(\begin{array}{c} 0\\ -\frac{1}{3}\omega r_{0}\alpha^{2}\cos\left(\omega t\right)\sin\left(\omega t\right)\\ 0 \end{array}\right) \end{array}.$$

The magnetic field from the attached magnet will have the form

(20) $$\begin{array}{c} B=\left(\begin{array}{c} B_{0}\sin\left(\alpha \sin\left(\omega t\right)\right)\\ B_{0}\cos\;\left(\alpha \sin\left(\omega t\right)\right)\\ 0 \end{array}\right) \end{array},$$

and the *z*-component of the resulting electric equilibrium field in the electroreceptor is

(21) $$E_{z}=\frac{1}{3}\left|B_{0}\omega r_{0}\alpha^{2}\cos\;\left(\omega t\right)\sin\left(\alpha \sin\left(\omega t\right)\right)\sin\left(\omega t\right)\right|.$$

For small values of *α*, we can use the approximation sin(*α*sin(*ωt*)) ≈ *α*sin(*ωt*) to get an expression for the z-component of the electric field, *E*_*z*_, induced by relative motion of the attached magnet:

(22) $$E_{z}\approx \frac{1}{3}B_{0}\omega r_{0}\alpha^{3}\text{si}{\text{n}}^{\text{2}}\left(\omega t\right)\cos\;\left(\omega t\right).$$

At typical swimming parameters (*ω* = 2*π*, *r*_0_ = 0.05 m) and assuming a small magnetic field (*B*_0_ = 0.02T), then relative angular movements with an amplitude greater than ≈0.107 radians (6.0 degrees) will cause an interfering signal at the vestibular frequency of 1 microvolt per meter.

This corresponds to a distance change between the magnet and the electroreceptor of ≈96.0 *μ*m during the swimming cycle.

## 6. Conclusions

We have presented, in Sections 2 and 4, a mechanism of induction-based magnetoreception based on measurements of electrosensory signals at harmonics of the vestibular frequency during swimming. Maintaining a constant swimming direction relative to the geomagnetic field could be accomplished by simply maintaining a constant electrosensory “chord”, consisting of different amplitudes at the harmonics of the vestibular frequency.

The analysis of signal amplitudes for some typical parameter values described in Section 3 shows that a magnetoreception mechanism based on measurements of induced electric fields at the harmonics of the vestibular frequency is plausible given the known detection thresholds for electroreceptor organs. The signals that are at harmonics of the vestibular frequency could be sensed by synchronous detection—correlation between electrosensory signals and vestibular signals.

We have also shown in Section 5 that a magnet placed in a flexible swimming fish would introduce strong signals at the vestibular frequency. Unless rigid criteria are met, these signals would interfere with an induction-based magnetoreception mechanism, and the experiments could not distinguish between induction and direct magnetoreception mechanisms. A simplified analysis showed that the body would have to be rigid enough to have no relative motion between the magnet and the electrosensory system within 100 *μ*m for this effect not to be present. As this criterion is unlikely to have been met in the previous experiments described by Hodson [12] and others [8], new experiments are needed to elucidate the sensory mechanism underlying magnetoreception in elasmobranch fishes.

### 6.1. Further Experiments

The analysis of Section 5 provides some guidance for further experiments that might differentiate between the induction-based and direct magnetoreception mechanisms. As the magnetic field of a magnet drops off rapidly with distance, an attached magnet with field strength a few times greater than the geomagnetic field would still interfere with a nearby direct magnetoreceptor but be weak enough that induced electrosensory signals are below sensitivity limits (due to both the weaker magnetic field and the reduced flexion of tissues over short distances).

For example, if an upper limit for the strength of the interfering electric field is chosen to be 0.01 microvolts per meter (this value is indicative only; any value well below the accepted thresholds for electrosensory sensitivity could be chosen) and the distance over which the magnetic field exceeds the geomagnetic field is measured to be 0.02 m, then at typical swimming oscillation frequencies (*ω* = 2*π*) and assuming a small magnetic field (*B*_0_ = 0.0001T), (22) shows that the induced electric field amplitude will be smaller than 0.01 *μ*Vm^−1^ if the relative angular movements have an amplitude less than ≈0.184 radians or 11.0 degrees. Experimental measurement of movement during swimming is required to confirm that relative angular movements exceeding 11.0 degrees do not occur between the magnet and the neighbouring tissues.

If orientation behaviour is not affected by such carefully chosen magnets, then the region surrounding the magnet would be excluded as a possible locus for a direct magnetoreceptive mechanism. Sufficient experimental coverage of plausible locations with fixed magnets would either exclude the direct magnetoreception mechanism entirely or provide good evidence for the location of a direct magnetoreceptor.

## Figures and Tables

**Figure 1:**
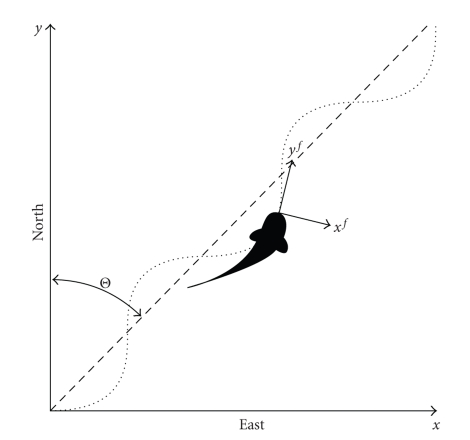
The stationary “earth” frame and the local body-frame (*x* ^*f*^, *y* ^*f*^) of the swimming fish, showing the heading angle Θ between magnetic north and the mean fish path.

**Figure 2:**
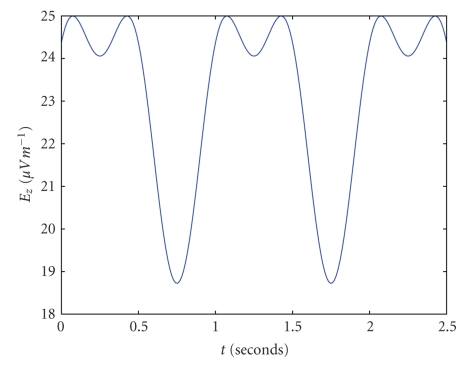
A plot of receptor electric field (in *μ*V*m* ^−1^) as a function of time for typical parameters. *B*_*y*_ = 2.5 × 10^−5^ T, *α* = 0.5, *ω* = 2*π*, *v* ^*w*^ = 1, and  Θ = 3*π*/7.

**Table 1:** Typical parameter values used when estimating the sensitivity required for electric navigation.

Parameter	Symbol	Typical value
Heading angle	Θ	45 deg
Horizontal magnetic field	*B*_*y*_	25 *μ*T
Vertical magnetic field	*B*_*z*_	25 *μ*T
Swimming speed	*v* ^*w*^	1 m s^−1^
Vestibular frequency	*ω*	2 *π* rad s^−1^
Angular modulation	*α*	0.5 rad (≈30 degrees)
